# The influence of knowledge of performance endpoint on pacing strategies, perception of effort, and neural activity during 30‐km cycling time trials

**DOI:** 10.14814/phy2.13892

**Published:** 2018-11-13

**Authors:** Georgia Wingfield, Frank Marino, Melissa Skein

**Affiliations:** ^1^ School of Exercise Science, Sport and Health Charles Sturt University Bathurst New South Wales Australia

**Keywords:** Central regulation, fatigue, pacing strategies, self‐paced exercise

## Abstract

It is understood that withholding information during exercise can alter performance during self‐paced exercise, though less is known about neural activity during such exercise. The aim of this study was to compare the effects of withholding versus providing distance feedback on perception, muscular activation, and cerebral activity during cycling time trials (TT). Nine well‐trained male cyclists randomly completed 2 x 30‐km TT, with provision of performance information and distance feedback (known; KTT), and without performance information and remaining distance (unknown; UTT). Prefrontal cortex (PFC) hemoglobin concentration, electroencephalogy (EEG) responses of the parietal lobe (PL) and motor cortex (MC), and surface electromyogram (EMG) of the right thigh were monitored throughout the TTs, in addition to heart rate (HR), rating of perceived exertion (RPE), and power output (PO). Time to completion was shorter for the KTT compared to UTT (51.04 ± 3.26 vs. 49.25 ± 3.57 min, *P* = 0.01). There were no differences evident for RPE between conditions (*P* > 0.50). However, during the final 2 km, the KTT presented higher PO (*P* ≤ 0.05), HR (*P* = 0.03) and MC, and PL EEG activity *(d *=* *0.51–0.71) in addition to increased tissue hemoglobin index (nTHI) and oxygen extraction (HHb) *(d *=* *0.55‐0.65) compared to the UTT. In conclusion, when withholding information pertaining to remaining distance, performance was reduced due to the application of a conservative pacing strategy. In addition, the increase in HHb across the PFC was strongly correlated with PO (*r* = 0.790; *P* < 0.001) suggesting knowledge about remaining distance may increase activation across the PFC. Further, it appears that changes within the PFC may play a role in the regulation of cycling performance.

## Introduction

While fatigue is normally considered a negative consequence of prolonged exercise, the specific mechanisms that regulate the fatigue process during endurance exercise remains somewhat elusive (Thomas et al. 2015). To date, the development of fatigue is suggested to be regulated by changes to central and peripheral components (Thomas et al. 2015), and thus, the regulation of “pace” during self‐paced exercise is proposed to be under the influence of conscious and subconscious control (Micklewright et al. 2016). Research further suggests that the subconscious regulation of pacing strategies may modulate intensity during an athletic event based on previous experience (Ulmer 1996) and afferent feedback (Amann and Dempsey 2008). Further evidence supports the notion that the subconscious regulation of exercise performance depends on the ability of the central nervous system (CNS) to distribute appropriate central motor drive to the active skeletal muscles (Amann and Dempsey 2008; Micklewright et al. 2010). As such, the commonly observed increase in power output (PO) at the end of a bout of self‐paced exercise, known as the end‐spurt, is associated with an increase in skeletal muscle recruitment (Gibson and Noakes 2004). Alterations to pacing strategies during an athletic event may be based on an internal calculation for energy requirements over the remaining duration of exercise, and in preparation for an “end‐spurt”, provided such information is available (Ulmer 1996; Ansley et al. 2004; Noakes 2012). However, if information pertaining to remaining duration is withheld, research has shown that athletes will utilize a more conservative, even pacing profile (Jones et al. 2013), including a slight decrease or more consistent work output for the entirety of the protocol (Billaut et al. 2011) while also reporting an increased perception of effort (Baden et al. 2005). While research relating to pacing profiles has been examined, much less is known about the neurological changes preceding and/or during these changes in exercise intensity, and the relationship between endurance performance, physiology, and perception of effort.

Increases in cardiovascular, thermoregulatory, and metabolic demands are synonymous with endurance exercise (Tucker and Noakes 2009), therefore, minimizing the perturbations associated with such demands will be valuable during prolonged exercise bouts. Centrally mediated responses to such physiological disruptions to homeostasis during self‐paced exercise are evidenced by changes in PO and alterations along the neural pathways (Noakes 2012; Stone et al. 2017). With methodological advances, evidence to support that the changes in skeletal muscle activation may be reflected by neural activity across the cerebral cortex is currently emerging. For example, Radel, Brisswalter, and Perrey (Radel et al. 2017) examined oxygenation of the prefrontal cortex (PFC) continuously during two 10‐min cycling trials, however, during one trial, participants were informed to cycle for 60 min and the test was stopped at 10 min. The authors reported that in anticipation of the prolonged exercise bout, there was a reduction in activity across the PFC for the duration of the trial despite no change in actual exercise duration. From their findings, these authors suggest the presence of strategic allocation of executive resources (i.e., attentional focus) was a coping mechanism in anticipation of a longer exercise duration. Additionally, Enders et al. (2016) propose that PO distribution may be initiated in the supplementary motor area of the frontal lobe during exercise to failure, indicating possible contributions to motor processing and planning. Collectively, these studies suggest that neural mechanisms may be involved in the strategic planning and executive function of cycling performance and the regulation of pacing strategies. While there is some evidence to suggest that changes in cerebral oxygenation during maximal exercise intensity are indicative of a reduction in motor unit recruitment (Robertson and Marino 2015) and the regulation of fatigue (Rupp and Perrey 2008), the role of the cerebral activity, in particular blood delivery and perfusion across the PFC during sub‐maximal, self‐paced exercise is not completely understood. Additionally, neural activity across the PFC and motor cortex (MC) are suggested to increase during the voluntary execution of motor activity (Suzuki et al. 2008) and during the final end‐spurt of an exercise trial (Billaut et al. 2010). However, there is little evidence to suggest that the regulation of such measures is dependent on the provision of performance feedback and remaining duration.

The relationship between physiological perturbations, exercise performance, and perceptual strain can be examined by manipulating and deceiving athletes of key exercise expectations including exercise intensity (Williams et al. 2016) and duration (Eston et al. 2012), knowledge of competitors (Corbett et al. 2012), inaccurate performance feedback (Stone et al. 2017), and/or the provision of remaining duration (Ansley et al. 2004). Rating of perceived exertion (RPE) is suggested to be in response to cumulative feedback from a number of physiological systems working within a theoretical template of performance output (Tucker and Noakes 2009; Noakes 2012), and is suggested to be an indication of afferent feedback (Tucker et al. 2006; Swart et al. 2009). Current findings indicate that when information about exercise endpoint is withheld (open‐loop), perception of effort is generated within the predetermined exercise template and is modified based on exercise intensity (Billaut et al. 2010), remaining duration (Hamilton and Behm 2017), previous experience (Micklewright et al. 2010), and physiological responses (Taylor and Smith 2017). However, due to varying exercise protocols, training status of participants and magnitude of deception experienced (Stone et al. 2017), results of these studies show that RPE may be increased (Williams et al. 2016), reduced (Eston et al. 2012), or remain unchanged (Billaut et al. 2011). As such, despite recent evidence suggesting RPE may play a role in modulating pacing strategies (Taylor and Smith 2017) and limit time to exhaustion (Salam et al. 2018) the development and continual adjustment of perceived exertion during self‐paced exercise, is yet to be confirmed.

Studies that have examined cerebral oxygenation across the PFC during strenuous exercise are mostly limited to examination of incremental tests to exhaustion (Rupp and Perrey 2008; Robertson and Marino 2015). To the authors’ knowledge, one study that has examined deoxygenated hemoglobin (HHb) concentration during self‐paced efforts reported a decrease in concentration was evident when participants voluntarily increased speed during an end‐spurt to completion; however, this study did not examine if this change was dependant on the knowledge of remaining duration (Billaut et al. 2010). Accordingly, the current study aims to examine changes in neural activity as determined by PFC hemoglobin concentrations and neural drive to the skeletal muscle while remaining time trial (TT) duration is withheld. Additionally, the current study aims to compare pacing strategies, physiological, and perceptual responses while withholding duration feedback during successive 30‐km cycling TTs. We hypothesize that with the provision of performance parameters and remaining duration, deoxygenated hemoglobin will be maintained until completion where increases will match increases in exercise intensity and EMG responses during an “end‐spurt” of PO due to additional consecutive planning across this region of the brain. We further hypothesize that when duration is withheld, participants will adjust pacing strategies to reflect a more conservative, even pacing profile for higher RPE values, and similar HR responses in an attempt to maintain metabolic resources due to the unknown remaining duration (Williams et al. 2014).

## Methods

### Participants

Nine competitive male cyclists volunteered to participate in this study. Participants trained an average of >150 km per week and were competing at the state and/or national level of competition (age 23.7 ± 6.6 years, mass 78.3 ± 5.7 kg, height 183.0 ± 4.6 cm, peak oxygen consumption (*V*O_2peak_) 63.6 ± 5.7 mL kg min^−1^). To determine *V*O_2peak_, participants performed a ramp test on a cycle ergometer (Wattbike Pro, Nottingham, United Kingdom), beginning at 120 W and increasing by 35 W every minute thereafter to exhaustion. Gas analysis was completed via a metabolic cart (Medgraphics Ultima System, Saint Paul). Consent was gained from the Institutional Human Research Ethics Committee prior to recruitment and all data collection.

### Overview

Following written and informed consent, participants completed a familiarization including a 30‐km time trial (TT) on an air‐braked stationary cycle ergometer (Velotron, RacerMate Inc., Seattle). During the familiarization, the participants were positioned to have a feedback of their performance in real time on a computer screen. Feedback included power output (PO), cadence, speed, time, distance, and heart rate (HR). The familiarization was to ensure test‐retest reliability of the 30‐km TT (Marino et al. 2002). Prior to commencing 30 km familiarization trial, participants completed 3 x 6 sec maximal effort sprints for normalization of the electromyography (EMG) data.

Participants then returned to the laboratories to complete a further two 30‐km TT in a randomized fashion. These trials were completed in 25.9 ± 2.3°C ambient temperatures. Following a 5 min low‐intensity warm‐up, participants completed maximal sprints from a low cadence start (<50 rpm). During sprints, participants were instructed and verbally encouraged to complete a maximal “all‐out” effort for 6 sec followed by 24 sec active recovery. Following 5 min active recovery from sprints, participants initiated the trial and were instructed to complete the 30 km as quickly as possible. Participants then completed either a known condition (known time trial; KTT) identical to familiarization including a 30‐km TT with feedback including HR, PO, cadence and speed, or the unknown (UTT) condition where all performance and exercise endpoint feedback was withheld for the duration of the trial. For standardization, bike set‐up, and gearing were consistent between conditions. During the KTT and UTT conditions, participants were permitted to observe their HR responses throughout the TT (F1, Polar, Electro‐Oy, Kempele, Finland). Prior to cycling trials, a mid‐stream urine sample was obtained to determine urine specific gravity (USG) as a measure of hydration status using a digital refractometer (PEN‐SW, Atago, Tokyo, Japan). If participants presented USG ≥ 1.020 mmol/L they were provided with 500 mL water to consume and USG was retested prior to commencing the warm‐up. Immediately pre‐ and post‐ cycling performance, capillary blood samples were taken from the fingertip of the nondominant hand for the assessment of glucose (Glu; Accucheck, Roche, Basel, Switzerland) and lactate (La^−^; Lactate Pro, Minneapolis) concentrations.

### Performance, physiological and perceptual measures

During maximal effort sprints, peak power output (PPO) was recorded as the highest PO in Watts (W) and during 30 km TT, mean PO was recorded as the average W during 30 sec epochs every 2 km. PO data were recorded by computer software integrated with the air‐braked cycle ergometer (Velotron CS 2008, RacerMate Inc., Seattle) and exported for later analyses (Excel 2007, Microsoft Corp, Washington). PO is also presented as a percent (%) change from peak sprint PO to provide relative comparisons to sprint EMG data. Resting HR was obtained after the participants were seated and rested for 5 min and then continuously recorded during TTs via chest‐strap and short‐range telemetry system (Polar Team Pro 2, Polar Elector‐Oy, Kempele, Finland). 30 sec epochs of HR was later analyzed over every 2 km during the 30‐km TTs. Ratings of perceived exertion (RPE) using the 6–20 point Borg RPE scale (Borg, 1982) were recorded every 2 km throughout the cycling protocol.

### Electromyography

Wireless Electromyography (EMG) electrodes (Trigno, Delsys, Boston, Mass) were placed onto the belly of right thigh including; vastus medialis (VM), rectus femoris (RF), vastus lateralis (VL), and biceps femoris (BF). The sites were prepared by abrasion then cleaned with alcohol wipes and skin was marked with a broad‐tip pen. For analyses, the data acquisition software (EMG Works, Delsys, Boston, Mass) was interfaced with LabChart (LabChart v8.1.6, ADInstruments, NSW, Australia) and simultaneously recorded throughout the duration of the cycling protocols. The EMG data were sampled at 1 kHz on the LabChart Software with a bandwidth filter of 20–450 Hz applied to remove movement artifact. The EMG data were later calculated as the root mean square (RMS) and analyzed using LabChart Reader (LabChart v8.1.2). At each 2 km interval during the TTs, every contraction during a 5 sec epoch were visually inspected for movement artifact, and the average 10 msec around the peak RMS amplitude of each contraction was examined. Peak EMG RMS during the maximal sprints was described as 100% recruitment. EMG data during the TT were normalized by dividing the peak EMG RMS of each 5 sec epoch every 2 km by the peak sprint values and are expressed as a percent (%) change.

### Electroencephalogram

The preparation and data acquisition of the Electroencephalogram (EEG) were followed based on manufacturer's instructions (B‐Alert, ABM, CA). A 20‐channel wireless EEG headset was fitted to the participants scalp, and the placement of electrodes based on the international 10–20 EEG system. EEG preparation and acquisition are consistent with those proposed in Robertson and Marino (2015). For examination of baseline EEG, participants remained in a seated position for 2 min with eyes open (EO) followed by 2 min with eyes closed (EC), all visual and noise distractions were removed. EEG measurements were also recorded for 90 sec (~1 km) every 3 km distance during each time trial. Data from channels C3, C4, P3, and P4 were used for analyses based on Brodmann's areas; motor cortex (MC: C3 and C4) and parietal lobe (PL; P3 and P4). All EEG data were processed and analyzed using the B‐Alert computer software (B‐Alert Lab, ABM, CA) where a decontamination process removed artifact including EMG and eye‐blink. Total power in alpha (*α*) frequency band‐waves (8–12 Hz) were examined as they are associated with wakefulness and mental readiness (Périard et al. 2018) and typically increase at the onset of exercise and are maintained during submaximal exercise intensities (Fumoto et al. 2010; Robertson and Marino 2015). All EEG data were later analyzed using Microsoft Excel then calculated as percent (%) change from baseline, EO.

### Near‐infrared spectroscopy

Concentrations of oxygenated (O_2_Hb) and deoxygenated (HHb) hemoglobin were measured at rest during baseline EEG‐EO, and continuously throughout all exercise trials via near‐infrared spectroscopy (NIRS) (NIRO‐200NX, Hamamatsu Phototonics, Shizouka, Japan). NIRS was examined by placing a light emitter and a light detector probe over the right hand side of the prefrontal lobe between Fp1 and F4 (international 10–20 EEG system). Sites were cleaned with alcohol wipes and probes were fixed via adhesive discs and secured with black velcro to deter additional light. The NIRS probes emit light at 775, 810 and 850 nm wavelengths which scattered light into the closely placed detector probes (interoptode distance = 4 cm). Beer‐Lamberts Law was internally implemented within the NIRO device to observe changes in O_2_Hb, HHb and is expressed as *μ*mol/L cm. Due to interference of superficial tissue layers, spatially resolved spectroscopy (SRS) parameters of the NIRO‐200NX system were applied to limit interference of superficial tissue during cerebral monitoring (Messere and Roatta 2013). Therefore, normalized tissue hemoglobin index (nTHI) was simultaneously recorded via SRS methods and is expressed in arbitrary units (a.u). The NIRS data were sampled at 1 sec and a 30 sec epoch was later analyzed at rest and every 2 km of each trial.

### Statistical analyses

Differences between conditions were analyzed using a two‐way (condition x time) repeated measures analysis of variance (ANOVA) for all variables collected at each interval including PO, HR, RPE, NIRS, EEG, and EMG. If significant differences were noted in the ANOVA, a protected LSD pairwise comparisons were conducted to establish where significant changes were present. To examine the magnitude of differences between conditions effect sizes (Cohen's *d*) were calculated and distributed into the following classifications; trivial (<0.2), small (0.2–0.49), moderate (0.5–0.79), and large (>0.80). In addition, to determine the strength of a relationship between exercise intensity and cerebral oxygenation, Pearson's product‐moment coefficients were conducted. Data are presented as mean ± standard deviation (SD). Significance was set at *P* ≤ 0.05. All data were analyzed using Statistical Package for Social Sciences (SPSS; Version 20, IBM corp., Armonk, NY).

## Results

### Performance

**Figure 1:**
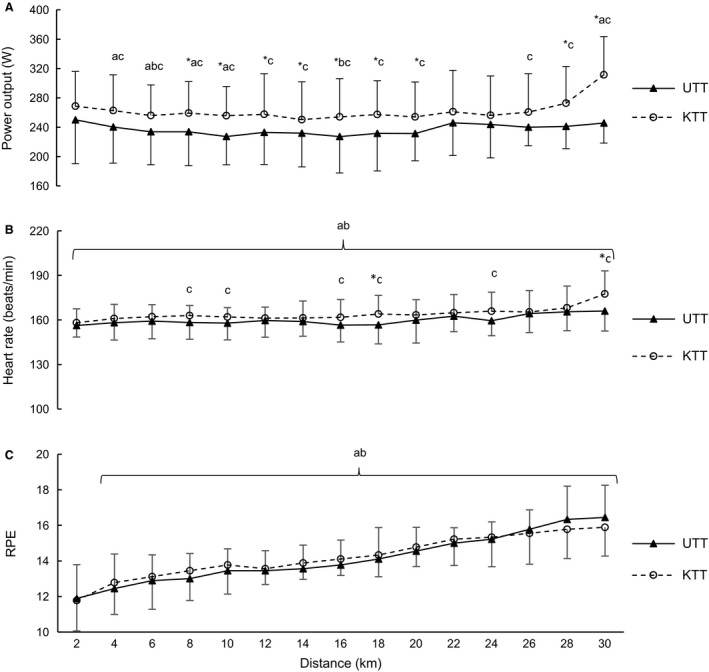
Mean ± SD for (A) Power output; (B) Heart rate; (C) Rating of perceived exertion (RPE) at 2 km intervals during the known (KTT) and unknown (UTT) 30‐km time trials (*n = *9); * Significant difference between UTT and KTT conditions (*P* = 0.00 – 0.05); ^a^Significant change overtime within the KTT condition (*P* = 0.001); ^b^Significant change overtime within the UTT condition (*P* = 0.01 – 0.04); ^c^Moderate to large effect size between KTT and UTT conditions (*d = *0.53–2.24).

There were no differences for PPO between the KTT or UTT conditions during the maximal effort sprints (KTT: 538 ± 103 W; UTT: 573 ± 90 W; *P* = 0.36). During the 30‐km TT, time to completion was significantly longer for the UTT condition compared to the KTT condition (UTT: 51.04 ± 3.26 min, KTT: 49.25 ± 3.57 min; *P* = 0.01). Figure 1A presents PO data during UTT and KTT conditions and shows that at 8–20, 28, and 30 km, PO was higher in the KTT compared to the UTT (*P* ≤ 0.05: *d *=* *0.65–2.24). Within the KTT condition PO was lower at 4–10 (*P* < 0.03) and higher at 30 km compared to the first 2 km (*P* = 0.001). While in the UTT condition, PO was lower at 6 and 16 km compared to the first 2 km (*P* = 0.04 and 0.01, respectively).

**Figure 2:**
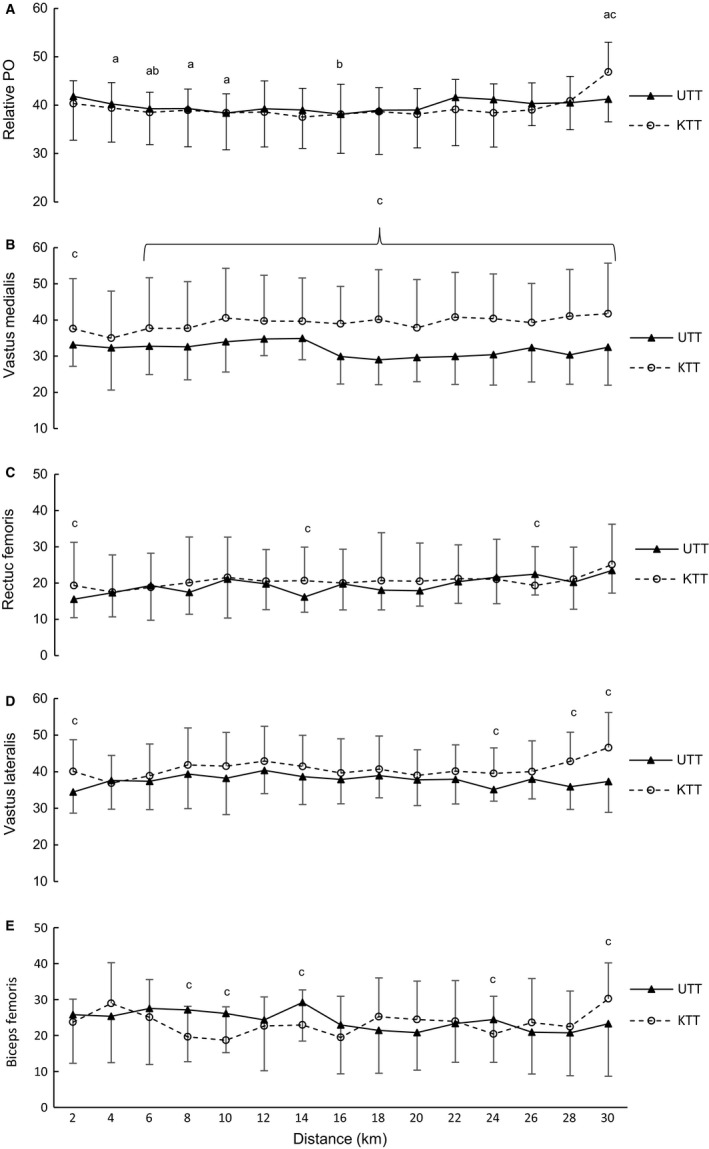
Mean ± SD percent change for (A) relative power output, (B) vastus medialis, (C) rectus femoris, (D) vastus lateralis, and (E) biceps femoris during the 30 km time trials for the known (KTT) and unknown (UTT) conditions (*n = *9); *Significant difference between UTT and KTT conditions (*P* = 0.04–0.05); ^a^Significant change overtime within the KTT condition (*P* = 0.03); ^b^Significant change overtime within UTT (*P* = 0.00 ‐ 0.03); ^c^Moderate to large effect size between KTT and UTT conditions (*d = *0.51–1.48).

Percent change from peak sprint PO (W) is presented in Figure 2A. There were no changes evident between the conditions (*P* > 0.06).

### Physiological and perceptual responses

As shown in Figure 1B, HR was higher for the KTT condition compared to UTT at 18 and 30 km (*P* = 0.03). Higher HR was also observed at all times compared to rest within both conditions (Resting HR: KTT: 61 ± 9; UTT: 61 ± 8; *P* = 0.001). Pre‐exercise USG showed no difference between the conditions (KTT: 1.017 ± 0.008, UTT; 1.014 ± 0.008; *P* = 0.51; *d *=* *0.45). Glu showed no change between or within UTT and KTT conditions at pre‐ and post exercise (KTT pre: 5.06 ± 0.6 and post: 5.2 ± 0.5 mmol/L; UTT pre: 5.6 ± 0.7 and post: 4.7 ± 1.3 mmol/L; *P* > 0.10; *d = *0.01–0.72). La^−^ was higher for KTT compared to UTT at postexercise (*P* = 0.05; *d = *1.45), and postexercise La^−^ was higher for both conditions compared to pre‐exercise (KTT pre: 1.8 ± 0.9 and post: 6.8 ± 1.9 mmol/L; UTT pre: 1.3 ± 0.2 and post: 5.0 ± 1.7 mmol/L; *P* < 0.001).

As shown in Figure 1C, no differences in RPE were evident between conditions (*P* > 0.50; *d *=* *0.09–0.45), however, RPE was higher within the KTT and UTT conditions from 4 to 30 km compared to the first 2 km (*P* = 0.00–0.02).

### Electromyography

There were no significant differences between conditions in EMG activity for VM, RF, VL, and RF (*P* > 0.05; Fig. 2). Effect size analyses revealed moderate effect of condition for VM activation at 2, 6, 8, 12, and 14 km (*d *=* *0.60–0.73) and large effects at 10 and 16–30 km (*d *=* *0.82–1.48). RF activity showed moderate effects between conditions at 2 and 26 km (*d *=* *0.51–0.58) and large effects at 14 km (*d = *0.88). VL EMG effects size analyses revealed large effect of condition at 2, 24, and 28–30 km (*d = *1.08–1.46). Finally, for BF EMG activity there was a moderate effect of condition at 24 and 30 km (*d = *0.51–0.79) and a large effect at 8, 10, and 14 km (*d = *0.86–1.04).

### Near‐infrared spectroscopy

**Figure 3:**
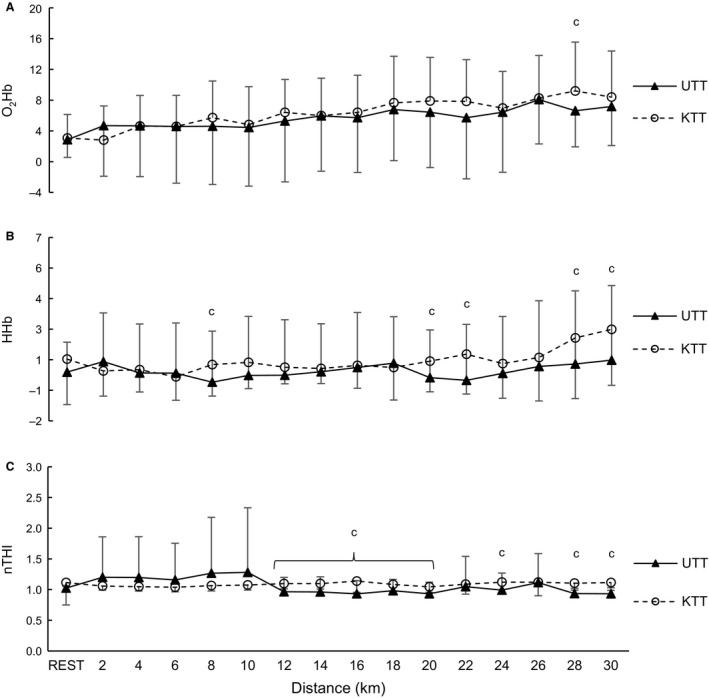
Mean ± SD of prefrontal hemoglobin concentrations for (A) oxygenated hemoglobin, (B) deoxygenated hemoglobin and (C) normalized tissue index in arbitrary units (a.u) during the 30‐km cycling trials for the known (KTT) and unknown (UTT) conditions (*n = *9); *Significant difference between UTT and KTT (*P* = 0.01–0.03); ^a^Significant change overtime within the KTT condition (*P* = 0.04–0.05); ^b^Significant change overtime within UTT (*P* = 0.03–0.05); ^c^Moderate to large effect size between KTT and UTT conditions (*d = *0.50–2.40).

Between the UTT and KTT conditions, there were no differences evident for O_2_Hb or HHb concentration or nTHI at any 2 km distance interval (*P* > 0.51; Fig. 3). However, there was a moderate effect of condition for O_2_Hb concentration at 28 km (*d *=* *0.65) and for HHb concentration at 8, 20, 22, 28, and 30 km (*d = *0.55–0.76; Fig. 3B). Further, normalized tissue hemoglobin index (nTHI) showed a moderate effect of condition at rest and 24 km (*d = *0.51–0.58) and large effects at 12–20 and 28–30 km (*d = *0.93–2.40; Fig. 3C).

The correlation coefficients between PO and any cerebral oxygenation concentration during the UTT condition were not significant (*P* ≥ 0.32). However, within the KTT condition, there was a strong relationship between HHb and PO (*r *=* *0.790; *P* < 0.001), while no other relationship within the KTT condition was evident (*P* > 0.29).

### Electroencephalogram

**Figure 4:**
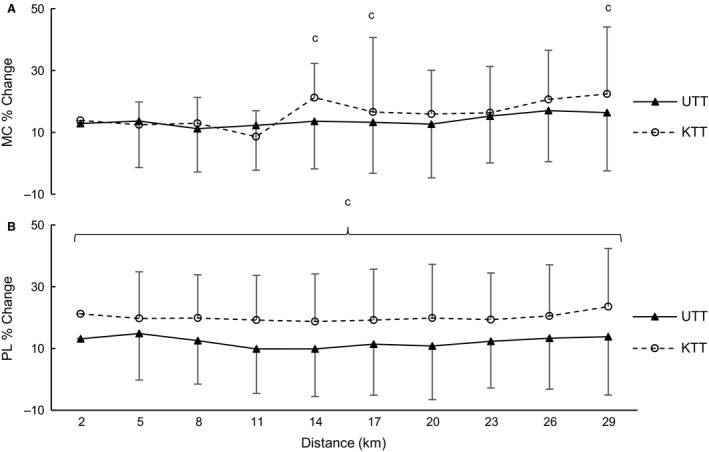
Mean ± SD of electroencephalogram (EEG) activity percent change from baseline (eyes open; EO) for (A) Motor Cortex; and (B) Parietal Lobe, during 30 km cycling trials during the known (KTT) and unknown (UTT) conditions (*n = *6). ^c^Moderate to large effect size between KTT and UTT conditions (*d = *0.50–0.87).

As shown in Figure 4, there were no differences between or within KTT and UTT conditions for EEG activity across the motor cortex (*P* > 0.07: Fig. 4A) or parietal lobe (*P* > 0.13; Fig. 4B). However, effect size analysis revealed a moderate effect of condition at the MC at 14 and 17 km (*d *=* *0.56 – 0.76) and at the PL at 4–8, 14–18, and 20–30 km (*d *=* *0.50–0.77) and large effects at the PL at 2, 12, and 18 km (*d = *0.82–0.87).

## Discussion

The primary aim of this investigation was to examine the effect of knowledge of versus blinding to exercise endpoint during 30‐km cycling TT on performance, pacing strategies, and the relationship with cortical activity. A novel finding in the current study was the higher normalized tissue hemoglobin index (nTHI) and deoxygenation (HHb) coinciding with an increase in PO during the characteristic end‐spurt for the KTT condition indicative of greater neural activity across the PFC. In addition, this study reports that when performance endpoint is available, participants were able to produce a higher PO throughout the trial and alter their pacing profile from a conservative even profile to allow for an end‐spurt, in spite of similar heart rate and RPE compared to withholding endpoint information. PO pacing profiles for both interventions coincided with EMG changes of the VM and VL muscles, however, these did not seem related to EEG activity across the motor cortex. Thus, withholding performance duration and exercise endpoint are associated with participants’ downregulating exercise intensity and engage in a more conservative pacing profile to accommodate for the unknown circumstances. Given the known role of the PFC in decision‐making and performance execution during exercise (Robertson and Marino 2016; Radel et al. 2017; Périard et al. 2018), these data from the present study provide evidence to suggest that the changes in deoxygenation across the prefrontal cortex may also play an important role in the formation and modification of an exercise template during self‐paced performances based on physiological feedback and knowledge of endpoint. Moreover, the PFC may contribute to the development and continual adjustment of rating of perceived effort during self‐paced exercise which can be influenced knowledge of exercise endpoint.

Typically, when participants engage in open‐loop exercise, pacing for optimal performance may be compromised as focus will shift to utilizing prior experience for completion of the task at hand, and minimizing homeostatic disturbance (Jones et al. 2013). As such, in the present study, when participates were provided with feedback regarding remaining distance to completion, they were able to sustain a higher PO for the duration of the 30‐km trial when compared to a trial where exercise duration was blinded. Interestingly, despite the differences in PO there were no changes between the conditions for HR until the final 2 km of the trials. As participants were provided with HR responses for the duration of both trials, there may have been pacing strategies adopted to regulate the rate of rise in HR, and thus, the down‐regulation of exercise intensity during the UTT condition may have been modified accordingly. Further, RPE was not different between the conditions, and while this follows the physiological perturbations (HR), others have shown that RPE is typically higher during open‐loop exercise (Billaut et al. 2011). Considering participants were in a similar physiological state when commencing exercise, and the difference in PO within the first 10 km of the trial, despite no differences in RPE, suggests that the mere lack of knowledge about information pertaining to exercise endpoint will alter pacing strategies to maintain a similar rating of perceived effort. This further highlights that the generation of RPE may be from feedback from range of sources, including the interpretation of exercise endpoint. Similar research has shown that open‐loop self‐paced exercise or blinding and deceiving participants of exercise duration, may divert the generation of pacing to be based on perception of effort (Paterson and Marino 2004), previous experience (Shei et al. 2016) and physiological feedback (Williams et al. 2016) rather than remaining duration. Specifically, evidence from Swart et al. (2009) shows that blinding cyclists to exercise endpoint until the final km of a 40‐km cycling TT produced a more conservative pacing profile and a perceived exertion strategy that would allow for a greater metabolic reserve. Therefore, it is likely that the reduction in PO and altered pacing profile in the present study may be the result of the unknown duration, coinciding with other physiological and psychological factors (Swart et al. 2009) to regulate exercise performance and maintain internal homeostatic conditions (Renfree et al. 2014).

There is still some debate regarding the regulation of drive to the muscle during endurance exercise, and whether fatigue can be attributed to changes at the periphery or modulated by central mechanisms (Decorte et al. 2012; Hureau et al. 2014). While it is suggested that activation of the working musculature is related to efferent drive (Amann and Dempsey 2008) research is yet to confirm the relationship between neural activity and muscle activation in response to open‐loop exercise (Billaut et al. 2011). In the current study, greater skeletal muscle activity was evident for the knee extensors during the KTT condition compared to the UTT condition. Further, the changes in activation and PO appear to be consistent up to the end‐spurt, which may suggest that muscle activation was increased in order to generate additional force for the final increase in PO, however, the antagonist muscle (BF) remains relatively unchanged. Research has shown that there is complexity in the patterns of co‐contraction between agonists and antagonists of EMG prior to fatigue (Hautier et al. 2000). Similarly, Hautier et al. (2000) reported no change in antagonist activity of the leg extensor muscles during repeat sprint efforts. The authors suggest this is primarily due to the higher training status of their participants and concluded that well‐trained cyclists can produce more efficiency during co‐contraction of pedaling action. The consistency in EMG activation and PO during the KTT condition is also reflected in the UTT condition, as there appears to be a steady EMG output for the duration of the UTT. This EMG output reflects the more conservative “even” pacing profile selected when participants were blinded to exercise duration. Research indicates that not only is pacing regulation based on sufficient prior experience of the exercise bout, but activation and tracking of active musculature to PO during such exercise are equally important and requires ample experience (Ansley et al. 2004). Therefore, we propose that the conservative pacing approach and reduction in activity of the skeletal muscle in the UTT condition are indicative of a reserve capacity (Stone et al. 2017) to reduce risk of premature fatigue during the unknown circumstances. Additionally, the increase in activation of the major muscles associated with the pedaling action reflected the increase in PO during the end‐spurt during the KTT condition, suggests participants in the KTT condition were able to anticipate and complete an end‐spurt by increasing neural drive to the appropriate musculature.

Pacing during self‐paced endurance events is supposedly premeditated based on previous experience and modified appropriately to remaining duration and current metabolic and psychological states, but further performance adjustments may occur in response to afferent feedback being interpreted by the brain (Subudhi et al. 2007; Marino 2014). As yet there is limited evidence to support this theory by examining changes in cerebro‐cortical activity. Upon examination of the current data, both conditions revealed an expected increase in cerebral oxyhemoglobin compared to rest (Shibuya et al. 2009), though no oxyhemoglobin differences were evident between conditions. The lack of difference between conditions may be due to the higher training status of the participants showing a greater ability to preserve the rate of oxygenated hemoglobin during exercise (Santos‐Concejero et al. 2014). Further, Rupp and Perrey (2008) have shown that a reduction in activity across the PFC, evidenced by reduced O_2_Hb concentration, becomes apparent upon reaching exhaustion at exercise intensities exceeding the respiratory compensatory point (RCP), near 80% *V*O_2max_ (Robertson and Marino 2015). The RCP is associated with increasing metabolic acidosis and minute ventilation (Broxterman et al. 2015), which creates a redistribution of blood flow (Querido and Sheel 2007) and is commonly followed by an immediate reduction in O_2_Hb prior to point of exhaustion (Perrey 2008). Further, recent evidence has eluded to the role of PFC in exercise termination during incremental exercise (Rupp and Perrey 2008; Robertson and Marino 2015). Given the self‐paced nature of the time trials in the current study, we suggest that the absence of a reduction in cerebral oxygenation during self‐paced work may be indicative of some regulation of oxygen delivery to eliminate the risk of developing hypoxia (Billaut et al. 2010). As exercise intensities throughout the TTs were below the RCP (in relation to the ventilatory data collected during baseline *V*O_2max_ testing), this may explain the lack of differences in O_2_HB and HHb observed during the first 26 km of the respective TTs. Moreover, the differences observed during the final 2–4 km between conditions may be the result of greater exercise intensities during the final end‐spurt in KTT. This regional increase in blood volume across the PFC coincides with efforts of high‐intensity exercise during the end‐spurt as participants are increasing exercise intensity approaching the RCP. Further, the current data demonstrate a strong relationship (*r *=* *0.790; *P* < 0.001) between oxygen extraction (HHb) and exercise intensity. There is evidence to suggest that the rate of deoxygenation during exercise may contribute to changes in exercise intensity to evade severe physiological harm by ischemia to vital organs (Billaut et al. 2010). Further, it has been proposed that while deoxygenation may occur during strenuous self‐paced exercise, it may be tightly regulated to maintain metabolic status, thereby influencing pacing strategies (Billaut et al. 2010). From these findings we suggest that an increase in the hemoglobin index may be indicative of some neural influence from the PFC over pacing strategies by regulating physiological reserve and rate of oxygen extraction during the KTT condition in preparation for the “sprint to the finish” (Billaut et al. 2010; Stone et al. 2017).

Unfortunately, due to data acquisition limitations, prefrontal cortex EEG data were not able to be processed; however, changes are apparent across the prefrontal cortex for oxyhemoglobin concentrations suggesting increased activity. Interestingly, despite the suggested changes in neural activity across the PFC, there were minimal differences between conditions for EEG alpha wave activity across the motor cortex. The role of the PFC at rest and during physical activity remains unclear, though there is some evidence to suggest that the PFC may execute functions associated with memory and decision‐making (Gallese and Lakoff 2005; Enders et al. 2016). Additional research has further suggested that the PFC may regulate exercise output by interpreting emotional and sensory information and feeding into the MC through afferent pathways (Robertson and Marino 2017). A novel finding in the current study is the lack of change across the motor cortex while a greater rate of oxygen extraction and hemoglobin index at the prefrontal cortex, and higher parietal lobe activity was evident, which corresponds with increasing exercise intensity. From these data, it may be suggested that the PFC may be more likely associated with the regulation of exercise intensity due to the role of interpreting emotion (Craig 2003) and the regulation of top‐down processing by guiding behavior (movement) based on internal states (afferent feedback) (Robertson and Marino 2015). In addition, activation of the primary sensory cortex within the parietal lobe is reported to be related to tasks involving attention and arousal (Coull 1998), and may communicate with the PFC (Edwards and Polman 2013). While traditional perspectives about the generation of central drive during incremental exercise to exhaustion comes from areas of the MC and premotor areas (Subudhi et al. 2009; Brümmer et al. 2011), we propose that during submaximal exercise, the PFC contributes to the generation of movement behavior based on sensory inputs. We further suggest that these data may indicate some cerebral regulation, particularly from regions of the PFC, over motor function in anticipation of the exercise endpoint during the KTT condition. These data highlight the importance of the PFC relating to the interpretation of emotions, internal state, and the moderation of pacing strategies during closed‐loop self‐paced exercise (Robertson and Marino 2015). Based on these findings, we suggest that the PFC may regulate physiological reserve during self‐paced exercise in anticipation of the end‐spurt when remaining duration is provided. Further, we also propose that the development of ratings of perceived effort may be based on collective feedback interpreted by this region of the brain to regulate exercise intensities and remain within physiological constraints (Craig 2003; Abbiss et al. 2015).

In conclusion, it appears that the provision of performance feedback and exercise endpoint can improve performance during self‐paced 30‐km cycling TTs. This improvement is achieved by an increase in skeletal muscle activation and allowing for higher PO during the end‐spurt to the finish. Further, our data suggest that there may be greater neural activity when knowledge of endpoint is available as made evident by increasing activity across regions of the PFC which may have contributed to the execution of the greater drive to the active muscles. These findings support the existence of a relationship between the brain and fatigue during self‐paced exercise, and further elude to the presence of physiological reserve during such exercise as metabolic resources were no longer at risk of disturbing homeostatic control during preparation for the sprint to the finish (Stone et al. 2017).

## Conflict of Interest

The authors declare no conflict of interest, financial, or otherwise.
